# Plant adaptation to cold climates

**DOI:** 10.12688/f1000research.9107.1

**Published:** 2016-11-25

**Authors:** Christian Körner

**Affiliations:** 1 Institute of Botany, University of Basel, Basel, Switzerland

**Keywords:** climate, development, freezing, growth, phenology, stress, biogeography

## Abstract

In this short review, I will first summarize criteria by which environments can be considered “cold”, with plant stature (size, height above ground) playing a central role for the climate actually experienced. Plants adapted to such environments have to cope with both extremes and with gradual influences of low temperature. The first requires freezing resistance, which is tightly coupled to developmental state (phenology) and prehistory (acclimation). Gradual low temperature constraints affect the growth process (meristems) long before they affect photosynthetic carbon gain. Hence, plants growing in cold climates are commonly not carbon limited.

## Introduction

Plant “adaptation” sounds like plants are “doing” something. In reality, the term subsumes a variety of adjustments, all rooted in evolution in the sense of having arrived in an “adapted state”, carrying adaptive traits. Some of these characteristics are genotypic ^1^, which means they are irreversible within the lifespan of a plant or several generations of plants (evolutionary adaptation). The second category of adjustments is the ability to modify plant structure during development, a response to the environment that is also not reversible within a plant’s or organ’s lifespan, which is often termed modification ^2^. Finally, some adjustments are reversible, and they are known as acclimation or physiological adjustments ^3^. Before we enter these various ways of being adapted, a brief account is needed on what is often termed “cold”.

## What is a cold environment?

It makes a big difference whether low temperatures prevail permanently or occur during certain parts of the year only (seasonally) or just diurnally, as in tropical mountains, with everyday summer and everyday winter; therefore, “cold” has a time and a latitude/elevation component. Further, the action of low temperature needs to be separated into gradual influences and extremes. There are cold or cool regions without severe freezing, permitting the growth of even some palm tree species (e.g. the city of Cork in Ireland), versus areas that experience, for instance, -38°C in winter but +43°C in summer (the city of Almaty in Kazakhstan) that require very high cold hardiness, despite the hot growing season, while the mean annual temperature (MAT) is almost the same at around +10°C. Quite obviously, a mean calculated across different seasons bears no ecological or biological meaning. Hence, MAT should be used with caution in any consideration of cold adaptation. As I will show, native plants have gone through regional selection and exhibit sufficient hardiness to survive the coldest extremes in their habitat in most cases.

In this overview, I refer to “cold” as free atmosphere conditions that deviate substantially (negatively) from optimum temperatures for growth in most vascular plant taxa on the globe, causing severe growth limitations (periodically no or very little growth for temperature reasons, or the risk of tissue damage and survival). Spatially, these are growing season conditions at high latitude (north of the polar circle) and high elevation (the climatic treeline and above), and, seasonally, these are cold periods in the temperate, boreal, and arctic life zones. For space reasons, I will disregard the effects of periodic low temperature limitations in otherwise warm and tropical climates, such as chilling damage.

Another problem is that we are used to describing “cold” conditions by adopting 2 m air temperature readings from meteorological stations rather than employing the actual temperatures experienced by plants. In reality, plants from cold environments can escape the action of free air circulation by reducing plant size and adopting compact growth forms, thereby engineering their own microclimate in addition to sheltered habitat selection. It has been shown that the actual temperatures experienced by plants during an alpine summer may differ as much from weather station data as if they were growing at 1500 instead of 2500 m elevation ^1, 2^. This is a consequence of the interaction of solar radiative heating with the aerodynamic layer properties around plant tissues ^3^. So we need to separate the action of extremely low temperatures from that of mean temperatures but also the temperatures reported from climate stations and those acting *in situ*. Alpine or arctic cushion plants' daytime canopy temperature may reach 25 to 30°C at an air temperature of around 8°C on a bright day. Similar solar warming may occur in the center of large alpine flowers ^4^. Throughout this article, I will be using °C for temperature and K (Kelvin) for temperature differences to avoid confusion between the two ^5^.

## Evolutionary adaptation

A cold-adapted genotype must, first of all, resist local low temperature extremes, so all plants found in cold climates have gone through that selection filter. Freezing resistance is the number one sieve that species must pass through to live in cold climates. The spectrum of freezing resistance reflects the spectrum of life conditions, and no plant can be found in this planet's periodically cold regions that is not resistant to the local low temperature extremes. This is not the place to review the knowledge on freezing resistance physiology (see 6, 7). In brief, freezing resistance in plants has little to do with antifreeze solutes. From physical chemistry, we know that 1 molar solution depresses the freezing point by 1.8 K only. Since the cells of most organisms are operating at or slightly below a 1 molar cell sap concentration, such a marginal gain in freezing point depression would require at least doubling concentration. Thus, the actual processes involved are water extraction from the cell through extracellular ice formation that requires a fluid outer cell membrane at low temperature, protective compounds such as certain sugars and proteins to safeguard membranes, and enzymes against damage during dehydration. For all this to work, the onset of extracellular ice formation must not be delayed, so that the flow of water out of the cell can keep pace with cooling. Surviving extremely low temperatures is the evolutionary outcome of cell membrane properties and certain cell sap constituents. Finally, freezing resistance is not constant for a given plant, but it varies, driven by internal controls (seasonality) and by short-term acclimation, all deeply rooted in evolution (phylogeny). In addition, some tropical alpine taxa have been found to perform what is called leaf super-cooling, which is retaining water in a gel-like metastable state below freezing point (to avoid nucleation). When the super-cooling capacity is exhausted around -12°C, tissues freeze immediately and are dead. There is evidence that the xylem water of trees of cold regions can super-cool down to -40°C (for a review, see 8).

The second important evolutionary adaptation to life under seasonally cold conditions is the fine-tuning of plant phenology. The timing of spring activity and autumnal senescence defines the actual length of the growing season. Phenology timing in spring prevents the exposure of active, vulnerable tissue to damaging low temperatures, but it also co-defines the remaining growing season length ^9^. Phenology controls also set the termination of growth activity in autumn before damaging freezing temperatures come into action. Plants adapted to cold climates must be able to complete their seasonal life cycle within the time frame set by phenology controls ^10^. During the dormant period, long-lived plants native to cold climates have gone through evolutionary selection and thus are commonly safe from fatal freezing damage; some even survive dipping in liquid nitrogen during the coldest part of the year. However, all of these plants may still experience some tissue loss during sharp frost events following warm spells or when obligatory snow bed plants become snow free (e.g. 11). In most cases, these losses can be compensated for by later regrowth.

The third evolutionary adaptation is plant stature. As mentioned above, the actual temperature experienced by plant tissues is strongly influenced by plant morphology. Small plant stature ensures a much warmer growing season than is experienced by trees, although small stature also enhances the risk of ground frost during clear nights, a fact particularly critical in the tropics ^12^. Tall stature enhances convective heat transfer and thus diminishes departures from air temperature. Hence, trees do not profit from any significant canopy warming during the day, the main reason for the formation of a high elevation or high latitude tree limit (the climatic treeline ^8^). In most alpine and arctic species, small stature is a genetic adaptation to low temperatures, and hence they stay small when grown in a warm environment. Plants (including tree species) may also be forced into small stature (become crippled) when they try to grow above the warm boundary layer near the ground, a modification that will not persist under warmer conditions. Yet, just like genetic dwarfs, phenotypic dwarfs benefit from the heat near the ground. This is why we find crippled trees (“krummholz”) above the climatic tree limit ^13^.

The net outcome of these three physiological and morphological adaptations is communities of cold-adapted species that can cope with the life conditions they experience. It is the absence of non-adapted species by which a given flora becomes cold adapted.

## Ecology of freezing resistance

The freezing resistance found in plants reflects their habitat type, their size, and their life expectancy. In trees, return rates of damaging temperatures of centuries or even millennia determine the presence of the species. In small-stature plants, the micro-environment near the ground influences success. That is why alpine snow bed plants exhibit comparatively low freezing resistance (they are well protected by snow in winter), whereas plants at locations with insecure snow cover must exhibit very high resistance ^14^. So, in alpine and arctic landscapes, freezing resistance follows the topography-controlled snow distribution patterns.

In trees, the most critical issue is spring and autumn phenology. The internal controls of spring flushing and autumnal hardening must not simply follow temperature because weather is intrinsically unreliable ^9, 15^. To safeguard plants against damage by flushing too early or hardening too late, they must employ reliable signals. A warm spell in late winter must not induce flushing, and in autumn senescence must be completed before the first critical freeze. Hence **,** trees with a long life expectancy commonly adopt a three-step control of spring flushing: for “knowing” that it is spring and not autumn (that winter has occurred), they measure chill degree hours by an unknown mechanism ^16, 17^. Once their chilling requirements are satisfied, they become sensitive to other signals. The effect of missing chilling can be seen by complete failure of winter wheat when sown in spring (no ears, just leaves). After having experienced sufficient chill, most late successional tree species switch from full dormancy (endodormancy) to “ecodormancy” and thus enter a phase during which photoperiod (a reliable, latitude-specific astronomic signal) and actual temperature interact. The closer the date at which the recurrence rate of species-specific damaging low temperatures becomes zero, the more thermal forcing can advance flushing. Early in spring, when severe frosts may still arrive, photoperiod constrains immediate responses to warm weather. The later it is in spring, the greater the influence of thermal forcing on development. This mechanism does not hold for exotic taxa (e.g. ornamental trees and some orchard trees). Such trees are tracking the warming weather at the wrong time and will be killed by late freezing events. While the risk of freezing damage diminishes with time in spring, it increases with time in autumn ^6, 18, 19^. That is why the transition to winter hardiness starts at a safe distance from the probable first incidence of critically low temperatures, and, to accomplish this, it is under photoperiod control. This primary induction becomes enhanced as trees experience chilling temperatures. In summary, the main control over freezing damage in cold-adapted trees is their phenology. In late successional trees, phenology tunes the balance between the need to escape late freezing events in spring and the need for a sufficiently long growing season (terminated by autumnal hardening and thus dormancy induction). As a result, trees are able to develop mature, winter-hardy tissues. The required minimum season length is determined by life history traits such as fruit size and wood anatomy ^10^.

## Growth and development in the cold

Cold-adapted plants need to be able to grow and pass through their seasonal and lifelong developmental steps under low, growth-constraining temperatures. There are principally two ways of achieving this: (1) use the warmest period only of an otherwise very cold climate (season length constraints) or (2) grow and develop under prevailing cool conditions. In high-latitude climates, the short growing season sets limits to (2); hence, other than in tropical high mountains, plants need to be fast as the season length shrinks. During their short active growing season (peak summer), conditions are commonly just as warm as at low elevation in spring, particularly in low-stature arctic and alpine plants. Not surprisingly, there is no thermal adjustment of photosynthesis to low temperature ^20^ because it is not cold at peak season and close to the ground during sunny days, yet it might get very cold during the night at high elevation in lower than high arctic latitudes. This leads to a syndrome of plant traits that include very fast development, high rates of metabolism, short tissue duration, and commonly substantial below-ground storage tissue. In contrast, in miserably cold but almost season-less climates in some wet tropical mountains, plants can compensate for low rates of metabolism by having long durations of activity. In both cases, the critical adaptation is the minimum temperature at which tissues can produce new cells. It was shown that no vascular plant can grow below 0°C, a temperature at which leaf photosynthesis may reach 30% of its full capacity. However, growth is commonly so slow below 5°C that the contribution of such hours to the seasonal biomass gain is negligible. This is why temperatures between 5 and 6°C have often been considered as the “zero point” of plant growth and development. It is not yet known which biochemical processes control that seemingly universal growth limit that holds from winter crops to treeline trees and glacier forefield plants ^21, 22^. Both cell wall synthesis and lignification are potential candidates. Some think it is the limitation of ATP production by mitochondria (e.g. 23, 24). Cold-adapted plants have increased the number of mitochondria in their cells ^25^; this makes them sensitive to periods of excessive warmth, causing respiration to overshoot. Super-cold-adapted plants have been found to exhibit a persistent Q _10_ of 3–4, instead of the common 2 (i.e. a doubling of respiration for a 10 K rise in temperature ^26^). A big respiratory machinery may be great in the cold, but it has its trade offs during warmer periods. These are the prime domains where future research of cold adaptation needs to engage.
